# Artificial intelligence in perioperative medicine education: A feasibility test of case-based learning

**DOI:** 10.1177/17504589251346634

**Published:** 2025-06-13

**Authors:** Timothy Trewren, Nicholas Fitzgerald, Sarah Jaensch, Olivia Nguyen, Alexander Tsymbal, Christina Gao, Brandon Stretton, Stewart Anderson, D-Yin Lin, Dario Winterton, Galina Gheihman, Guy Ludbrook, Kelly Bratkovic, Stephen Bacchi

**Affiliations:** 1 Department of Anaesthesia, Adelaide Medical School, The University of Adelaide, Adelaide, SA, Australia; 2 Flinders Medical Centre, Bedford Park, SA, Australia; 3 Harvard Medical School, Boston, MA, USA; 4 Lyell McEwin Hospital, Elizabeth Vale, SA, Australia; 5 Flinders University, Bedford Park, SA, Australia

**Keywords:** Large language models, Machine learning, Medical education, Natural language processing, Case-based learning, Artificial intelligence

## Abstract

The use of artificial intelligence in medicine is rapidly expanding. Large language models, such as ChatGPT, have the potential to enhance perioperative medicine through education and clinical practice. However, concerns remain regarding the accuracy of these models, particularly the risk of hallucinations, generating factually incorrect outputs. This feasibility test explores the use of a large language model–enabled platform to assist in case-based education in perioperative clinical cases.

**Methods:**

Five perioperative cases addressing core topics were developed and uploaded to a custom large language model platform. The large language model platform allows free-text questions to be asked to the artificial intelligence, which then uses the derived cases to provide answers. Anaesthetic trainees engaged with the artificial intelligence, asking questions to obtain information regarding history, examination, and investigations. Artificial intelligence question-and-answer pairs were then evaluated independently in duplicate for the presence of inappropriate responses, including hallucinations.

**Results:**

The large language model responded appropriately to nearly all questions, with no hallucinations observed. The proportion of questions that were answered appropriately was 99.3% (543/547). In the four instances of inappropriate responses, the large language model declined to provide information in the case description rather than hallucinate.

**Conclusion:**

The large language model appears capable of supporting the delivery of case-based perioperative medicine content with a high degree of accuracy.

## Introduction

Artificial intelligence (AI) has the potential to significantly impact perioperative medicine and healthcare professional education (Chan et al 2025). Large language models (LLMs), such as the widely known ChatGPT, could increase the interactivity of medical education through chatbot interfaces. One such circumstance in which interactivity could be improved with LLM is case-based learning (Stretton et al 2024). However, concerns regarding the veracity of the presented information remains, particularly in a healthcare education setting.

AI medical education perioperative applications occur in the broader context of AI, increasingly being applied to preoperative risk stratification, intraoperative decision support and postoperative outcome prediction. For example, machine learning algorithms that ingest large perioperative data sets can forecast major complications and mortality with greater accuracy than traditional scores, enabling clinicians to tailor and improve management based upon individual risk (Ren et al 2022). Similarly, real-time deep-learning monitors have also been shown to effectively detect nociceptive events and hemodynamic instability during surgery (Abdel Deen et al 2025). Multiple narrative reviews now map how AI methods stand to reshape every phase of perioperative medicine (Maheshwari et al 2023, Nathan 2023).

LLMs are rapidly being explored for medical education. Systematic reviews indicate that LLM chatbots can supply personalised explanations, generate practice questions, and simulate clinical scenarios for both undergraduate and postgraduate learners (Li et al 2024, Xu et al 2024). Classroom studies further suggest that interacting with an LLM tutor improves both learner engagement and perceived preparedness for future clinical encounters (Zhou et al 2025).

LLMs, however, are prone to generating distorted ‘hallucinations’. Hallucinations are plausible sounding but factually incorrect outputs. In evaluations of scientific-writing tasks, ChatGPT fabricated references in up to 30% of outputs (Chelli et al 2024). Likewise, medical trainees show limited ability to detect hallucinated clinical details in LLM-generated answers (Zhou et al 2025). Any educational deployment must therefore incorporate expert oversight, authoritative grounding, explicit instruction on limitations of the technology, and a robust scientific evaluation.

Previous research has suggested that LLMs can be effectively used to deliver case-based content in medical specialties like neurology (Gim et al 2025). However, it has also been shown that LLMs may underperform in medical domains that are less well represented in presumed training corpora (Asiedu et al 2024). Given the developing nature of the field of perioperative medicine, it is uncertain whether LLMs would be more likely to underperform in this specialty.

Therefore, the aims of this study were to explore the accuracy of AI-generated responses, and to record any problematic hallucinations when cases were delivered in an interactive question-and-answer format with LLMs from curated case descriptions.

## Materials and methods

### Overview

**Figure 1:**
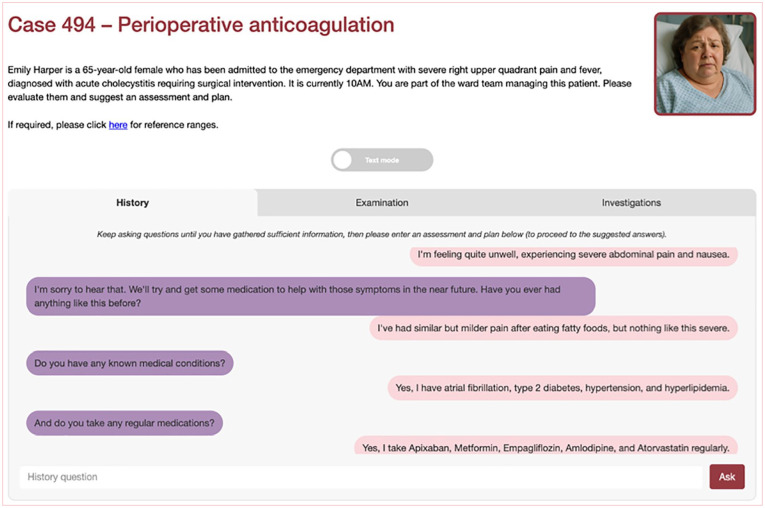
Online platform demonstrating history

**Figure 2:**
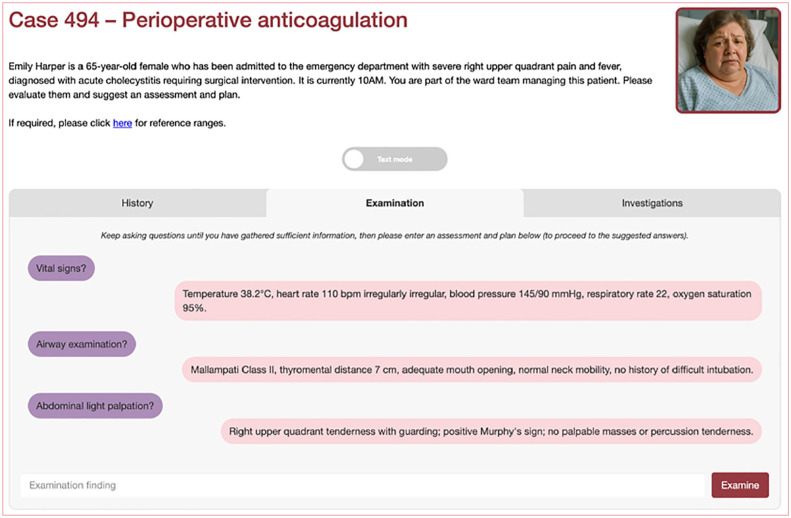
Online platform demonstrating examination

**Figure 3:**
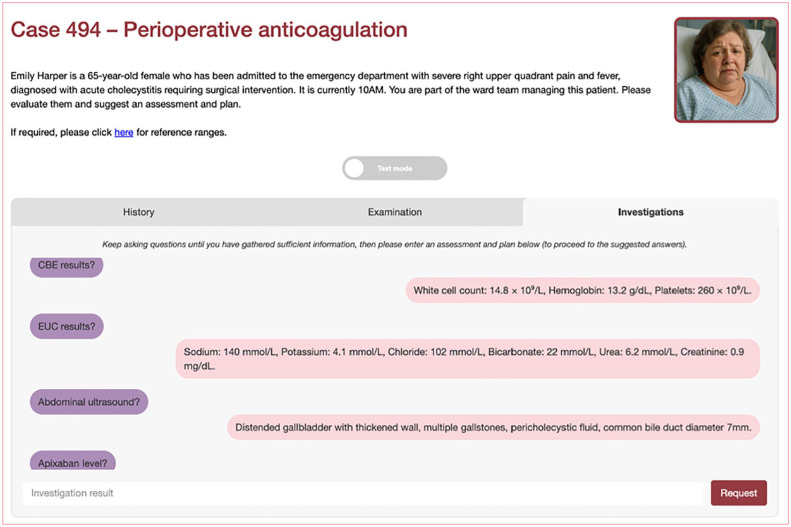
Online platform demonstration investigations The images in Figures 1–3 are original and were created by the authors for the purposes of this manuscript. They are derived from the online LLM platform through which the investigators interacted with. They have not been sourced from a commercial or third-party provider.

Case descriptions (‘screenplays’) were developed using LLMs, prior to being edited by trainees and medical education consultants. They were subsequently delivered through an online LLM-enabled platform (see Figures 1–3). These cases were then questioned by investigators, and the question-and-answer response pairs evaluated for hallucinations in duplicate.

### Case generation

Five perioperative cases were produced for this study. The five topics selected were based on medical student curricula pertaining to perioperative management issues and included anticoagulation management, fluid management, insulin management, prophylactic antibiotics, and being nil by mouth with time critical medications (e.g. medications for Parkinson’s disease). Case outlines were generated using the LLM Claude 3.7 Sonnet from Anthropic. The prompts that were used to generate these cases were based on the structure included in Supplementary Information 1.

After cases were generated by the LLM, they were reviewed by an intensive care unit trainee and a medical education consultant. This process involved independently reading the cases and manually editing aspects that were felt to be incongruent with medical practice, or adding content that was felt likely to be required in the real-world evaluation of such patients. The finalised screenplays that were used are included in Supplementary Information 2.

### Case delivery

When the screenplays were finalised, they were uploaded to a custom online interface. This online platform provides the entire screenplay to a second LLM (GPT-4o) which then enables users to interact with it in a question-and-answer format as if they were evaluating a patient (or in physical examination and investigations, requesting them from another clinician). This second LLM was used both because of developer familiarity and its performance based on trends in sycophancy and online leaderboard rankings at the time of development.

Four anaesthetic trainee investigators then independently evaluated each of the cases with questions as if they were evaluating a patient. An unlimited number of questions and time was allowed, with the intent to evaluate each case until they had sufficient information to develop an assessment and plan. These investigators all had the opportunity to trial the online platform on at least one case prior to evaluating the perioperative cases in this study.

### LLM evaluation

Question-and-answer pairs were then downloaded from the online platform and evaluated. This evaluation was conducted in duplicate by the intensive care unit trainee and medical education consultant who developed the screenplays. This evaluation included reviewing each investigator question and associated LLM answer, and categorising the response in a number of ways, namely:

(a) whether the LLM answered the question (with an example of a non-response being ‘I don’t have that information at the moment’) and, if it declined, if this was an appropriate response (i.e. if the information was not present in the screenplay, then declining to answer would be considered appropriate);
(b) if the question was answered whether the answer was on the screenplay (as opposed to an unanticipated question);
(c) if the question was not on the screenplay, to which part of the patient evaluation did the question refer (i.e. history, examination, or investigations);
(d) if the answer was not on the screenplay, and the LLM provided a response (i.e. ‘made up’ new content), was the content medically congruent and reasonable (i.e. if the answer was no, then this would be a problematic hallucination).

Following categorisation, question-answer-pairs were evaluated with descriptive statistics. The primary outcome was the proportion of medically appropriate LLM answers to investigator questions. This is the converse of the number of problematic ‘hallucinations’ or inappropriately declined answers. We deemed hallucinations in this study to be defined as unfounded fabrication and or factual errors. Unfounded fabrication describes instances where the system generates information, such as facts, data, or opinions, without sufficient evidentiary support or reliable references. Factual errors involve incorrect or inaccurate representations of objective information or empirical data presented in the system’s output (Sun et al 2024).

### Ethical approval

This study involved the evaluation of a publicly available LLM using investigator-generated content and did not require ethics approval. The project was undertaken as an educational innovation initiative to explore the feasibility of LLM-assisted case-based learning in perioperative medicine. As such, it aligns with institutional guidelines for educational improvement activities that do not constitute human research.

## Results

There were a total of 547 question-and-answer pairs obtained in the study. This equates to a mean of 136.8 questions per investigator who undertook the cases, and a mean of 109.4 questions per case.

Overall, the proportion of questions that were answered appropriately was 99.3% (543/547). All instances of inappropriate answers were instances in which the LLM did not provide information that was available in the screenplay when asked. In all four of these questions, the LLM instead inappropriately declined with a response similar to ‘This information is not available’. The questions pertained to results from an electrocardiogram, ketone levels, a patient’s height and weight, and evidence of injuries to aspects of the patient other than that requiring surgery.

With respect to possible hallucinations, there were 0% (0/547) responses that involved the introduction of inappropriately fabricated content. There were 14.4% (79/547) questions in which the LLM answered content that was not present in the screenplay. In other words, the LLM introduced or added content beyond that which was in the screenplay. In these questions, and in every instance, the information generated was medically appropriate. Examples of these instances are summarised in Table 1, with a complete list available in Supplementary Information 3.

**Table 1:** Examples of answers provided by LLM that were not included in screenplay

Case	Question	Answer	Category	Explanation*
Perioperative anticoagulation	How many years did you smoke for?	I smoked for about 20 years before quitting.	History (previous smoking duration)	Described 20 year smoking history, which is reasonable.
	When did you last take empagliflozin and metformin?	I last took Empagliflozin and Metformin at 8:00 PM yesterday.	History (previous metformin dose timing)	Gave same time for last dose of metformin as the last dose of empagliflozin and apixaban, which is reasonable.
	Any caps or crowns?	I don’t have any dental caps or crowns.	History (dental)	Described no caps or crowns on teeth in history. The physical exam aspect of screenplay describes one crown. The LLM would not have had access to that part of the screenplay when providing history. This was deemed medically reasonable as patients may well forget previous dental procedures, and examination would be required to supplement this history.
	What is usual BSL	I usually keep my blood sugar levels between 100 and 150 mg/dL.	History (usual BGLs)	Described regular BGL range
	How do you manage your diabetes	I manage my diabetes with Metformin and Empagliflozin, along with a healthy diet and regular monitoring.	History (diet and exercise)	Described diet and exercise that were not included in screenplay.
Perioperative fluid balance	How bad is your pain currently out of 10	My pain is about 8 out of 10 right now.	History (pain score)	Described 8 out of 10 pain for fracture.
	Have you had any pain relief	I haven’t taken any pain relief yet since my fall.	History (analgesia received)	Described not having received analgesia, which is improbable, but also a patient may not recall, and it is possible.
	Is your cardiologist happy with your heart	Generally, my cardiologist has been monitoring my conditions, but recent symptoms have raised concerns.	History (cardiologist progress)	Been more concerned recently
Perioperative antibiotics	Have you ever had cephalexin or cephazolin	I haven’t taken either cephalexin or cephazolin before.	History (antibiotic exposure)	While not specifically told about other antibiotic exposure, it responded reasonably without declining to have the information.
Perioperative glucose	What doses of insulin do you use	I use 30 units of insulin glargine at night and 8 units of insulin lispro with meals.	History (medication dosage)	Doses not provided in screenplay, reasonable dose provided.

* In this study the LLM was specifically encouraged to add details to aspects of the history if they did not directly influence the core issues at hand. This creation of content was a desired feature to improve the fidelity of the educational experience, with fewer history questions being declined. For example, a patient responding with ‘No information is available’ regarding diet, would seem more incongruous than a similar response to a request for a particular blood test. The definition of a problematic hallucination used in this study was as described in the method section, namely unfounded fabrications. Therefore, when the LLM successfully generated content that was medically appropriate, despite not being in the ‘screenplay’ this was desired and not considered a problematic hallucination. The explanation column describes why the investigators reviewing these comments deemed the content to be medically reasonable, and hence not an ‘unfounded fabrication’.

## Discussion

These results suggest that the LLM can be used to accurately represent written case descriptions in an interactive question-and-answer format for learners. There was a very low rate of inappropriate responses, and when they did occur, it was due to the lack of provision of information by the LLM. Importantly, there were no instances of inappropriate hallucination.

An important distinction in this study is the use of LLMs in an educational setting, and not for clinical decision making. In this educational context, ‘unfounded fabrication’ merited classification as an inappropriate hallucination. Given the educational focus of portraying a patient case, plausible generation of content beyond that contained in the screenplay was not only acceptable, but designed and desired. For example, in the history section, the LLM was deliberately deployed in a manner to allow it to generate plausible content that did not change core content relating to the medical issues in the case. This utilisation of the generative components of LLM may allow for more fluent case interactivity and were not adjudicated as hallucinations if medically appropriate.

This educational application is in sharp contrast to clinical applications of LLMs. If a LLM were applied to a clinical setting, such as scribe applications and audit activities, the generation of plausible content (that was encouraged in this educational setting) could be directly harmful to patient care or unit activities (Goh et al 2024, Kleinig et al 2024). In such a clinical context, any production of plausible content not explicitly described would be considered an ‘unfounded fabrication’. At the core of this distinction lies the intent of the LLM application (i.e. providing a realistic educational patient case vs completing a clinical task such as documentation), and this highlights the importance of use-case specific LLM deployments and prompting. Clinical applications of LLMs require different structures, verification methods, and benchmarking to those in educational settings like that in this study.

This study adds perioperative medicine to the growing list of disciplines in which LLMs can accurately deliver interactive case-based learning. Our zero-hallucination rate in this sample of five cases when the model was anchored to a curated screenplay parallels the high factual concordance reported when ChatGPT answered standardised medical examinations (Kung et al 2023) and supports findings that careful prompt design and content grounding can curb error generation in educational settings (Zhou et al 2025).

Educationally, AI-driven virtual cases can furnish unlimited, on-demand deliberate practice while standardising exposure to core perioperative scenarios. Reviews of LLM use in medical training highlight benefits such as individualised feedback, improved engagement, and scalability to resource-limited settings (Xu et al 2024). Embedding such tools into curricula could therefore extend experiential learning beyond operating-room schedules and faculty availability, expanding traditional education practices to enhance readiness for an evolving healthcare landscape (Cadman et al 2025). However, scientific evaluation of these tools is necessary before widespread deployment.

If LLMs are not used carefully, problematic hallucinations can still occur and responses may perpetuate historical biases or race-based misconceptions present in training data (Omar et al 2025, Omiye et al 2023). The opaque reasoning of transformer models further complicates error detection and accountability. Ongoing supervision, routine content validation, and bias-mitigation strategies are therefore essential before widescale educational adoption. The findings of this study support the existence of approaches that could enable the delivery of such AI supported content for healthcare professional education with negligible hallucinations.

The interactivity of LLMs demonstrated through this online educational platform also raises the potential for similar interfaces as a tool to support clinical activities. The nature of hallucinations and LLM deployment in clinical settings differs to that of the demonstrated educational setting. For example, the creation of information on diet and exercise in case 1 (anticoagulation), in a clinical setting could mislead triage process for prehabilitation, and inaccurate information on insulin dose regimens could lead to inappropriate insulin prescription. Preliminary studies in the clinical domain show encouraging results with similar online interfaces, enabling the collection of information as a medical officer may conduct (Gao et al 2025). Future evaluation of LLMs for clinical applications will require different methods of evaluation to this type of educational application. For example, there is currently an increasing focus on the evaluation of clinical applications of LLMs as similar to that of the credentialling of medical professionals (Rajpurkar & Topol 2025). The potential of LLMs to assist in collection of data from patients, and in assisting decision making, in an era of shortages of medical personnel, is significant, but in such clinical applications the eradication of hallucinations will be vitally important.

As a feasibility analysis, this research has several limitations. While five cases spanning multiple perioperative topics is a strength, generalisability would be further supported by the inclusion of more, and more diverse, cases. In addition, determining the incidence of events in the absence of an observed event is challenging, and accurate estimates would be supported by such future studies. Furthermore, all cases were written and evaluated in English. While this study has demonstrated that the content can be delivered successfully with this method, improvement in educational outcomes have not yet been demonstrated.

To integrate AI into medical education for perioperative patient assessment and management plan formulation, further research is needed to align AI with existing teaching methods. AI may enhance adult learning by fostering problem-solving and decision-making case scenarios rather than just delivering content (Mehigan et al 2023). In the future, AI cases can be linked to specific curriculum objectives, providing personalised feedback on students’ patient assessments and management plans, which could support targeted skill development. For clinicians unfamiliar with AI, this approach may contextualise the technology within familiar educational frameworks. Combining AI with traditional methods, such as in-person learning, would create a blended environment that enhances education across diverse learning styles, supporting deliberate practice in clinical decision-making and patient care (Sameen et al 2022).

The role of LLM in tasks adjacent to the delivery of question-and-answer case-based content should also be explored further, including the ability of the models to provide learners with feedback and automated scoring (Qian et al 2025). Now we have shown accuracy of an LLM with no hallucinations in an interactive case delivery, determined by detailed assessment by expert clinicians, we can further assess accuracy within a blinded clinical review format to develop a more robust assessment of the model.

## Conclusion

LLMs, a type of AI, can be used to successfully deliver case-based perioperative medicine content based on a high degree of accuracy. This has the potential to provide high-quality learning with low reliance of human input. Further studies examining the effect of such resources, including the future use in supporting clinical care, are required.

## Supplemental Material

sj-docx-1-ppj-10.1177_17504589251346634 – Supplemental material for Artificial intelligence in perioperative medicine education: A feasibility test of case-based learning Supplemental material, sj-docx-1-ppj-10.1177_17504589251346634 for Artificial intelligence in perioperative medicine education: A feasibility test of case-based learning by Timothy Trewren, Nicholas Fitzgerald, Sarah Jaensch, Olivia Nguyen, Alexander Tsymbal, Christina Gao, Brandon Stretton, Stewart Anderson, D-Yin Lin, Dario Winterton, Galina Gheihman, Guy Ludbrook, Kelly Bratkovic and Stephen Bacchi in Journal of Perioperative Practice

## References

[bibr1-17504589251346634] Abdel Deen OMT Fan S-Z Shieh J-S 2025 A multimodal deep learning approach to intraoperative nociception monitoring: Integrating electroencephalogram, photoplethysmography, and electrocardiogram Sensors 25 1150 40006379 10.3390/s25041150 PMC11859842

[bibr2-17504589251346634] Asiedu M Tomasev N Ghate C et al 2024 Contextual evaluation of large language models for classifying tropical and infectious diseases [Online] Available from: https://ui.adsabs.harvard.edu/abs/2024arXiv240909201A [Accessed April 2025].

[bibr3-17504589251346634] Cadman V Batty H Law J 2025 Implementation of research, education and leadership placements into Operating Department Practitioner training: A 4-pillar practice-based learning approach Journal of Perioperative Practice 35 183–188 39396125 10.1177/17504589241276743 PMC12008462

[bibr4-17504589251346634] Chan LKM Mao BP Zhu R 2025 A bibliometric analysis of perioperative medicine and artificial intelligence Journal of Perioperative Practice 4 17504589251320811 10.1177/17504589251320811 40035147

[bibr5-17504589251346634] Chelli M Descamps J Lavoué V et al 2024 Hallucination rates and reference accuracy of ChatGPT and Bard for systematic reviews: Comparative analysis Journal of Medical Internet Research 26 e53164 10.2196/53164 PMC11153973 38776130

[bibr6-17504589251346634] Gao C Bellinge J El-Masri S et al 2025 Not such a long way off? Contemporary artificial intelligence performance evaluation on adult medicine ‘long cases’ medRxiv [Online] Available from https://www.medrxiv.org/content/10.1101/2025.01.25.25321118v1

[bibr7-17504589251346634] Gim H Cook B Le J et al 2025 Large language model-supported interactive case-based learning: A pilot study Internal Medicine Journal 55 852–855 40125598 10.1111/imj.70030

[bibr8-17504589251346634] Goh R Cook B Stretton B et al 2024 Large language models can effectively extract stroke and reperfusion audit data from free-text discharge summaries Journal of Clinical Neuroscience 129 1108847 10.1016/j.jocn.2024.110847 39305548

[bibr9-17504589251346634] Kleinig O Gao C Kovo JG et al 2024 How to use large language models in ophthalmology: From prompt engineering to protecting confidentiality Eye (Lond) 38 649–653 37798360 10.1038/s41433-023-02772-w PMC10920651

[bibr10-17504589251346634] Kung TH Cheatham M Medenilla A et al 2023 Performance of ChatGPT on USMLE: Potential for AI-assisted medical education using large language models Public Library of Science Digital Health 2 e0000198 10.1371/journal.pdig.0000198 PMC9931230 36812645

[bibr11-17504589251346634] Li J Dada A Puladi B et al 2024 ChatGPT in healthcare: A taxonomy and systematic review Computer Methods and Programs in Biomedicine 245 108013 38262126 10.1016/j.cmpb.2024.108013

[bibr12-17504589251346634] Maheshwari K Cywinski JB Papay F et al 2023 Artificial intelligence for perioperative medicine: Perioperative intelligence Anesth Analg 136 637–645 35203086 10.1213/ANE.0000000000005952

[bibr13-17504589251346634] Mehigan S Cenarosa AS Smith R et al 2023 Engaging perioperative students in online learning: Human factors Journal of Perioperative Practice 33 4–8 36062457 10.1177/17504589221107227 PMC9827490

[bibr14-17504589251346634] Nathan N 2023 Perioperative artificial intelligence Anesthesia & Analgesia 136 636 36928148 10.1213/ANE.0000000000006427

[bibr15-17504589251346634] Omar M Sorin V Agbareia R et al 2025 Evaluating and addressing demographic disparities in medical large language models: A systematic review International Journal for Equity in Health 24 57 40011901 10.1186/s12939-025-02419-0 PMC11866893

[bibr16-17504589251346634] Omiye JA Lester JC Spichak S et al 2023 Large language models propagate race-based medicine Nature Partner Journals Digital Medicine 6 195 10.1038/s41746-023-00939-z PMC10589311 37864012

[bibr17-17504589251346634] Qian C Gao C Park S-O et al 2025 Use of large language models for rapid quantitative feedback in case-based learning: A pilot study Medical Science Educator Epub ahead of print 28 February. DOI: 10.1007/s40670-025-02343-6. PMC12228618 40625928

[bibr18-17504589251346634] Rajpurkar P Topol EJ 2025 A clinical certification pathway for generalist medical AI systems Lancet 405 20 39755382 10.1016/S0140-6736(24)02797-1

[bibr19-17504589251346634] Ren Y Loftus TJ Datta S et al 2022 Performance of a machine learning algorithm using electronic health record data to predict postoperative complications and report on a mobile platform JAMA Network Open 5 e2211973 10.1001/jamanetworkopen.2022.11973 PMC9112066 35576007

[bibr20-17504589251346634] Sameen Z Talib K Wani SQ et al 2022 Preoperative education improves the preparedness for extubation at emergence from general anaesthesia Journal of Perioperative Practice 32 41–46 32648835 10.1177/1750458920936213

[bibr21-17504589251346634] Stretton B Kovo J Arnold M et al 2024 ChatGPT-based learning: Generative artificial intelligence in medical education Medical Science Educator 34 215–217 38510403 10.1007/s40670-023-01934-5 PMC10948641

[bibr22-17504589251346634] Sun Y Sheng D Zhou Z Wu Y 2024 AI hallucination: Towards a comprehensive classification of distorted information in artificial intelligence-generated content Humanities and Social Sciences Communications 11 1278

[bibr23-17504589251346634] Xu T Weng H Liu F et al 2024 Current status of ChatGPT use in medical education: Potentials, challenges, and strategies Journal of Medical Internet Research 26 e57896 10.2196/57896 PMC11391159 39196640

[bibr24-17504589251346634] Zhou J Zhang J Wan R et al 2025 Integrating AI into clinical education: Evaluating general practice trainees’ proficiency in distinguishing AI-generated hallucinations and impacting factors BMC Medical Education 25 406 40108629 10.1186/s12909-025-06916-2 PMC11924592

