# Supplementary material for: Artificial intelligence in perioperative medicine education: A feasibility test of case-based learning
Source: J Perioper Pract. 2025 Jun 13;36(1-2):50–7. doi: 10.1177/17504589251346634 (PMC12712223; doi:10.1177/17504589251346634)
Supplement: sj-docx-1-ppj-10.1177_17504589251346634 – Supplemental material for Artificial intelligence in perioperative medicine education: A feasibility test of case-based learning [file sj-docx-1-ppj-10.1177_17504589251346634.docx]

**Supplementary Information**

**Table of Contents**

[Supplementary information 1 – Case generation prompt structure 16](#_Toc196304898)

[Supplementary Information 2 – Case descriptions provided to LLM (“screenplays”) 20](#_Toc196304899)

[Anticoagulation case 20](#_Toc196304900)

[Introduction 20](#_Toc196304901)

[Case 20](#_Toc196304902)

[Key points to elicit 23](#_Toc196304903)

[Explanation 23](#_Toc196304904)

[Assessment and plan exemplar 23](#_Toc196304905)

[Necessary points for assessment and plan 23](#_Toc196304906)

[Fluids case 24](#_Toc196304907)

[Introduction 24](#_Toc196304908)

[Case 24](#_Toc196304909)

[Key points to elicit 26](#_Toc196304910)

[Explanation 26](#_Toc196304911)

[Assessment and plan exemplar 26](#_Toc196304912)

[Necessary points for assessment and plan 27](#_Toc196304913)

[Insulin case 28](#_Toc196304914)

[Introduction 28](#_Toc196304915)

[Case 28](#_Toc196304916)

[Key points to elicit 30](#_Toc196304917)

[Explanation 30](#_Toc196304918)

[Assessment and plan exemplar 31](#_Toc196304919)

[Necessary points for assessment and plan 31](#_Toc196304920)

[NBM medications case 32](#_Toc196304921)

[Introduction 32](#_Toc196304922)

[Case 32](#_Toc196304923)

[Key points to elicit 34](#_Toc196304924)

[Explanation 34](#_Toc196304925)

[Assessment and plan exemplar 35](#_Toc196304926)

[Necessary points for assessment and plan 35](#_Toc196304927)

[Antibiotic prophylaxis case 36](#_Toc196304928)

[Introduction 36](#_Toc196304929)

[Case 36](#_Toc196304930)

[Key points to elicit 38](#_Toc196304931)

[Explanation 38](#_Toc196304932)

[Assessment and plan exemplar 38](#_Toc196304933)

[Necessary points for assessment and plan 38](#_Toc196304934)

[Supplementary Information 3 – LLM Responses not in screenplays 39](#_Toc196304935)

# **Supplementary information 1 –** Case generation prompt structure

You are a medical education expert. You are going to write a demonstration case (targeting interns) with the issue of a patient who has been admitted with XX. They have a past medical history that includes XX. The case is to describe the evaluation and management plan of the team on the floor (ward) prior to the patient undergoing an operation. The patient’s name is XX and is XX-years-old. Please change parts of the text and add investigations and examination findings as required. However, please keep all the same sections. Please ensure the Key points to elicit and Assessment and Plan exemplar concise - no more than 4-5 brief bullet points in each section. Use simple phrases rather than complete sentences (same for Key points to elicit, which should include key investigation result). Avoid specific medication dosages, detailed scheduling, or extended explanations in the plan. The Assessment should list only the key diagnoses without detailed descriptors, and the Plan should outline only the essential management steps without elaboration. Here is an example cases showing the exact format and style required:

**Introduction**

Mrs. Roberta Hale is a 78-year-old female who has been admitted to the emergency department following a fall at home resulting in a neck of femur fracture.

**Case**

**HISTORY**

**Name:** Mrs. Roberta Hale
**Age:** 78
**Gender:** Female
**Home:** Lives alone in a single-story apartment
**Handed:** Left-handed
**Occupation:** Retired nurse
**Drives:** No, uses community transport
**Activities of daily living:** Requires assistance with shopping and cleaning
**Past medical history:** Moderate aortic stenosis (diagnosed 5 years ago), hypertension, type 2 diabetes mellitus, osteoporosis
**Regular medications:** Alendronate 70mg weekly, Metformin 500mg twice daily, Perindopril 5mg daily, Atorvastatin 20mg daily
**Allergies:** Penicillin (rash)
**Smoking:** Never smoked
**Alcohol:** Occasional glass of wine (1-2 standard drinks per week)
**Recreational drugs:** Nil
**Over the counter medications:** Calcium and vitamin D supplements daily
**Herbal medications:** Nil
**Vaccinations:** Up to date with influenza, pneumococcal, and COVID vaccines
**Sexual history:** Widowed 10 years ago
**Family history:** Mother had osteoporosis, father died of stroke at age 82

**PRESENTING CONCERN**

**Chief complaint:** Left Hip Pain After Fall

**History of presenting complaint:** I was walking to the bathroom in the early morning when I felt dizzy and lost my balance. I fell onto my left side and couldn't get up because of severe pain in my left hip. I managed to reach my medical alert device and call for help. The paramedics found me on the floor about an hour after my fall. I've been having mild dizziness for the past few weeks, especially when standing up quickly. I've also noticed I get short of breath when walking more than half a block or doing light housework. Sometimes I feel a fluttering sensation in my chest that lasts for a few minutes. I have chronic pain in both knees from arthritis, which makes walking difficult. I haven't had any recent fevers, changes in vision, or headaches. My appetite has been poor lately, and I've lost about 5 pounds over the past three months without trying.

**NPO STATUS**

**Last oral intake:**

- Solid food: Dinner at 6:00 PM yesterday (soup and crackers)
- Clear liquids: Water at 5:00 AM today **Duration of fasting:** 16 hours for solids, 5 hours for clear liquids at time of assessment

**ANESTHETIC HISTORY**

**Previous anesthesia experiences:** General anesthesia for cholecystectomy (age 55), local anesthesia for cataract surgery (age 70)
**Complications with anesthesia:** Post-operative nausea after cholecystectomy, no other complications
**Family history of anesthetic complications:** None known

**SPECIFIC EVALUATION FOR ANESTHESIA**

**Metabolic equivalents (METs) capacity:** cannot walk half a block without symptoms

**MEDICATION RECONCILIATION**

**Specific timing of last dose of antihypertensives:**

- Perindopril 5mg: Taken at 8:00 PM yesterday

**EXAMINATION**

**Vital signs:**

- Temperature 37.0°C (98.6°F)
- Heart rate 92 bpm
- Blood pressure 145/85 mmHg
- Respiratory rate 20 breaths per minute
- Oxygen saturations 94% on room air
- Blood glucose level 155 mg/dL (8.6 mmol/L)

**AIRWAY ASSESSMENT**

**Mallampati classification:** Class III
**Thyromental distance:** 6 cm
**Mouth opening and dentition:** Adequate mouth opening (2.5 finger breadths), partial upper dentures, lower dentition with multiple missing teeth
**Neck mobility:** Limited extension due to age-related changes
**History of difficult intubation:** None reported

**Mental status:** Alert and oriented to time, place, and person. Glasgow Coma Scale 15/15.

**General inspection:** Elderly female, appears uncomfortable and in pain. Mild pallor noted.

**Cardiovascular examination:**

- Regular rate and rhythm
- JVP not elevated
- 3/6 mid-systolic ejection murmur, loudest at right upper sternal border
- Normal S1, soft S2
- No S3 or S4
- Mild bilateral ankle edema
- Peripheral pulses present but diminished

**Respiratory examination:** Bibasal crackles, otherwise clear

**Abdominal examination:** Soft, non-tender, no organomegaly

**Neurological examination:**

- Cranial nerves intact
- Power 5/5 in upper limbs, unable to assess lower limbs due to pain
- Normal tone and reflexes
- Plantar responses downgoing
- No cerebellar signs

**Musculoskeletal examination:**

- Left lower limb externally rotated and shortened
- Severe pain on minimal movement of left hip
- Tenderness over left greater trochanter

**Height:** 160 cm **Weight:** 68 kg **BMI:** 26.6 kg/m²

**INVESTIGATIONS**

**Blood tests**

- Complete blood examination: Hemoglobin, blood: 11.8 g/dL [Reference Range: 12.0-15.5 g/dL]
- EUC: Creatinine: 1.2 mg/dL (106 μmol/L) [Reference Range: 0.5-1.1 mg/dL]
- Troponin T: 16 ng/L [Reference Range: <14 ng/L]
- BNP: 120 pg/mL [Reference Range: <100 pg/mL]
- Coagulation studies:
  - PT: 13.2 seconds [Reference Range: 11.0-13.5 seconds]
  - INR: 1.2 [Reference Range: 0.8-1.2]
  - aPTT: 32 seconds [Reference Range: 25-35 seconds]
- Calcium: 8.8 mg/dL [Reference Range: 8.5-10.5 mg/dL]
- Vitamin D (25-OH): 22 ng/mL [Reference Range: 20-50 ng/mL]

**Electrophysiology and functional testing**

- ECG: Sinus rhythm at 92 bpm. Left ventricular hypertrophy by voltage criteria. No acute ST-T wave changes.

**Imaging**

- X-ray left hip: Displaced fracture of the femoral neck, Garden classification III
- Chest X-ray: Mild cardiomegaly. No pulmonary edema or consolidation.
- Transthoracic Echocardiogram: Moderate aortic stenosis (AVA 1.2 cm², peak gradient 48 mmHg, mean gradient 30 mmHg, velocity 3.5 m/s). Left ventricular hypertrophy. Preserved left ventricular ejection fraction (55%). Mild mitral regurgitation. No regional wall motion abnormalities.

**Key points to elicit**

(1) Displaced femoral neck fracture (2) Moderate aortic stenosis (3) Recent dizziness and exertional dyspnea (4) Mild anemia (Hgb 11.8 g/dL) (5) Elevated BNP (120 pg/mL) (6) Limited neck mobility (7) Multiple comorbidities (T2DM, HTN, osteoporosis)

**Explanation**

This patient presents with a displaced femoral neck fracture following a fall likely precipitated by postural hypotension in the setting of moderate aortic stenosis. Her cardiac status is of significant concern for anesthetic planning, with moderate aortic stenosis, mild cardiomegaly, and elevated BNP suggesting some degree of cardiac decompensation. The patient's symptoms of exertional dyspnea and dizziness over recent weeks suggest worsening of her cardiac status. Her airway assessment reveals potential difficulty with a Mallampati Class III and limited neck mobility.

The surgical urgency of a hip fracture must be balanced against optimization of her cardiac status. Early surgery (within 24-48 hours) is generally recommended for hip fractures to reduce complications, but careful anesthetic planning is essential given her cardiac risk factors.

**Assessment and plan exemplar**

**Assessment:**

**Displaced femoral neck fracture**

**Moderate aortic stenosis, symptomatic**

**Mild anemia**

ASA Class: III (Severe systemic disease with functional limitation)

**Plan:**

- IV fluid careful rehydration
- Cardiac optimization
- Cardiology consult
- Spinal anesthesia if feasible
- Hemodynamic monitoring
- ICU bed request

**Anticoagulant/antiplatelet medications and timing:**

- None regular

**Other medications to continue/hold perioperatively:**

- Recommend holding Perindopril day of surgery
- Metformin to be held 24 hours before surgery
- Alendronate not due during perioperative period
- Calcium and vitamin D supplements can be continued

**Necessary points for assessment and plan**

(1) Displaced femoral neck fracture (2) Moderate aortic stenosis (3) Anemia (4) Cardiology consultation (5) Anesthetic technique (spinal preferred) (6) Hemodynamic monitoring (7) Post-operative ICU

Please write the new case in this style.

# **Supplementary Information 2** – Case descriptions provided to LLM (“screenplays”)

##

## Anticoagulation case

### Introduction

Mrs. Emily Harper is a 65-year-old female who has been admitted to the emergency department with severe right upper quadrant pain and fever, diagnosed with acute cholecystitis requiring surgical intervention. It is currently 10AM. You are part of the ward team managing this patient. Please evaluate them and suggest an assessment and plan.

### Case

HISTORY

**Name:** Mrs. Emily Harper
**Age:** 65
**Gender:** Female
**Home:** Lives with husband in a two-story house
**Handed:** Right-handed
**Occupation:** Retired teacher
**Drives:** Yes
**Activities of daily living:** Independent
**Past medical history:** Atrial fibrillation, type 2 diabetes mellitus, hypertension, hyperlipidemia
**Regular medications:** Apixaban 5mg twice daily, Metformin 1000mg twice daily, Empagliflozin 10mg daily, Amlodipine 5mg daily, Atorvastatin 40mg daily
**Allergies:** Sulfa drugs (rash)
**Smoking:** Ex-smoker (quit 15 years ago)
**Alcohol:** Occasional (2-3 glasses of wine per week)
**Recreational drugs:** Nil
**Over the counter medications:** Multivitamin daily
**Herbal medications:** Nil
**Vaccinations:** Up to date with influenza, pneumococcal, and COVID vaccines
**Sexual history:** Married 40 years
**Family history:** Father had coronary artery disease, mother had breast cancer

PRESENTING CONCERN

**Chief complaint:** Right Upper Quadrant Pain
**History of presenting complaint:** I woke up two days ago with severe pain in my right upper abdomen. The pain is constant, sharp, and worse with movement or deep breathing. I've had some nausea and vomited twice yesterday. I've had no appetite since the pain started. I've noticed my urine is darker than usual, and my husband mentioned my eyes look yellow. I've had a fever with chills since yesterday. I've had similar but milder pain after eating fatty foods in the past few months, but it usually went away. I haven't had any recent changes in bowel habits. I've been taking my usual medications, including my blood thinner for my heart rhythm problem.

NPO STATUS

**Last oral intake:**

- Solid food: Light breakfast (toast) at 7:00 AM yesterday
- Clear liquids: Sips of water at 6:00 AM today **Duration of fasting:** 25 hours for solids, 4 hours for clear liquids at time of assessment

ANESTHETIC HISTORY

**Previous anesthesia experiences:** General anesthesia for appendectomy (age 20), spinal anesthesia for knee arthroscopy (age 58)
**Complications with anesthesia:** Post-operative nausea after appendectomy, no other complications
**Family history of anesthetic complications:** None known

SPECIFIC EVALUATION FOR ANESTHESIA

**Metabolic equivalents (METs) capacity:** can climb one flight of stairs without symptoms

**STOPBANG assessment for OSA**: occasionally snores, no daytime tiredness, no observed apnoea’s, known to have hypertension, BMI 27.5 as below, neck assessment as below, gender female

**History of GORD or reflux symptoms:** does not experience any symptoms of reflux normally, but has been having some mild reflux since the pain started.

MEDICATION RECONCILIATION

**Specific timing of last dose of anticoagulants:**

- Apixaban 5mg: Taken at 8:00 PM yesterday
- Empagliflozin last taken at 8pm yesterday

EXAMINATION

**Vital signs:**

- Temperature 38.2°C
- Heart rate 110 bpm, irregularly irregular
- Blood pressure 145/90 mmHg
- Respiratory rate 22 breaths per minute
- Oxygen saturations 95% on room air
- Blood glucose level 9.2 mmol/L

AIRWAY ASSESSMENT

**Mallampati classification:** Class II
**Thyromental distance:** 7 cm
**Mouth opening and dentition:** Adequate mouth opening (3 finger breadths), good dentition with crown on upper right molar
**Neck mobility:** Normal range of motion
**History of difficult intubation:** None reported

**Mental status:** Alert and oriented to time, place, and person. Glasgow Coma Scale 15/15.
**General inspection:** Middle-aged female, appears uncomfortable and in pain. Mild jaundice noted in sclera.

**Cardiovascular examination:**

- Irregularly irregular rhythm
- JVP not elevated
- No murmurs, normal S1 and S2
- No S3 or S4
- No peripheral edema
- Peripheral pulses present and equal
- Warm and flushed peripherally
- Central capillary refill 2 second, peripherally 1 second

**Respiratory examination:** Clear bilaterally, no crackles or wheezes
**Abdominal examination:**

- Right upper quadrant tenderness with guarding
- Positive Murphy's sign
- Percussion tenderness absent
- No palpable masses
- Normal bowel sounds
- No renal angle tenderness

**Neurological examination:**

- Cranial nerves intact
- Power 5/5 in all limbs
- Normal tone and reflexes
- Plantar responses downgoing
- No cerebellar signs

**Height:** 165 cm **Weight:** 75 kg **BMI:** 27.5 kg/m²

INVESTIGATIONS

**Blood tests**

- Complete blood examination:
  - White cell count: 14.8 × 10⁹/L [Reference Range: 4.0-11.0 × 10⁹/L]
  - Hemoglobin: 13.2 g/dL [Reference Range: 12.0-15.5 g/dL]
  - Platelets: 260 × 10⁹/L [Reference Range: 150-400 × 10⁹/L]
- Liver function tests:
  - Total bilirubin: 2.8 mg/dL [Reference Range: 0.1-1.2 mg/dL]
  - Direct bilirubin: 1.6 mg/dL [Reference Range: 0.0-0.3 mg/dL]
  - ALT: 125 U/L [Reference Range: 7-55 U/L]
  - AST: 98 U/L [Reference Range: 8-48 U/L]
  - ALP: 210 U/L [Reference Range: 40-150 U/L]
  - GGT: 180 U/L [Reference Range: 9-48 U/L]
- EUC:
  - Sodium: 140 mmol/L [Reference Range: 135-145 mmol/L]
  - Potassium: 4.1 mmol/L [Reference Range: 3.5-5.0 mmol/L]
  - Chloride: 102 mmol/L [Reference Range: 98-107 mmol/L]
  - Bicarbonate: 22 mmol/L [Reference Range: 22-29 mmol/L]
  - Urea: 6.2 mmol/L [Reference Range: 2.5-7.5 mmol/L]
  - Creatinine: 0.9 mg/dL (80 μmol/L) [Reference Range: 0.5-1.1 mg/dL]
- Coagulation studies:
  - PT: 14.8 seconds [Reference Range: 11.0-13.5 seconds]
  - INR: 1.3 [Reference Range: 0.8-1.2]
  - aPTT: 34 seconds [Reference Range: 25-35 seconds]
  - Apixaban level: 50 [Reference Range: 30-412]
- HbA1c: 7.2% [Reference Range: <6.5%]
- CRP: 95 mg/L [Reference Range: <5 mg/L]
- VBG:
  - pH 7.30 [Reference Range: 7.35-7.45]
  - CO2: 35 [Reference Range: 35-45]
  - Lactate: 2.2 [Reference Range: <1.0]
  - BGL: 9.2mmol/L [Reference Range: 6-10]
- Ketone: 1.6

**Electrophysiology and functional testing**

- ECG: Atrial fibrillation with ventricular rate 110 bpm. No acute ischemic changes.

**Imaging**

- Abdominal Ultrasound: Distended gallbladder with thickened wall (6mm). Multiple gallstones with largest measuring 1.5cm. Pericholecystic fluid. Common bile duct diameter 7mm (upper limit of normal).
- Chest X-ray: No acute abnormalities. Normal cardiac silhouette. No pulmonary edema or consolidation.

### Key points to elicit

(1) Acute cholecystitis (2) apixaban most recent dose 8pm (3) Empagliflozin most recent dose 8pm

### Explanation

This patient presents with acute cholecystitis, confirmed by clinical, laboratory, and imaging findings. Management is complicated by concurrent atrial fibrillation requiring anticoagulation with apixaban, and type 2 diabetes mellitus. The surgical urgency of acute cholecystitis must be balanced against the bleeding risk associated with recent apixaban use. The patient shows signs of systemic inflammation with leukocytosis, elevated CRP, and fever, suggesting progression of the inflammatory process.

The mild elevation in bilirubin and common bile duct at the upper limit of normal raise concern for potential common bile duct obstruction, though there is no frank biliary obstruction at this time. The patient's diabetes management is also important to optimize perioperatively, with her current HbA1c indicating suboptimal control.

### Assessment and plan exemplar

**Assessment:** Acute cholecystitis

Atrial fibrillation on apixaban

Type 2 diabetes mellitus

Evolving euglycaemic ketoacidosis secondary to empagliflozin use

ASA Class: III

**Plan:**

- IV fluids – further fluid resuscitation would be warranted pre-operatively given the pathology is causing evolving sepsis which may progress to septic shock, her lactate is above the normal range, and the euglycaemic ketoacidosis requires ongoing fluid management
- Antibiotics – guided by local guidelines, but empirical therapy may consist of Piperacillin/Tazobactam OR Ceftriaxone +/- Metronidazole
- Hold apixaban, ideally for 48 hours before surgery. If unable, consider discussion with haematology. Timing of recommencement to be guided by surgical progress.
- Hold empagliflozin and monitor. Monitor ketones and BGLs
- Initiate an insulin infusion with glucose to manage the ketosis and prevent further metabolic acidosis pre-operatively
- Planning for laparoscopic cholecystectomy – consideration of induction agents and early initiation of vasopressor therapy to prevent significant haemodynamic instability
- Continue nil by mouth with only sips of water until theatre

### Necessary points for assessment and plan

(1) Acute cholecystitis (2) IV fluids (3) Antibiotics (4) Hold apixaban (5) Hold empagliflozin and metformin (6) Ketone and blood glucose monitoring

## Fluids case

### Introduction

Mrs. Grace Donovan is an 81-year-old female who has been admitted to the emergency department following a fall at home resulting in a neck of femur fracture. It is currently 12PM (midday). You are part of the ward team managing this patient. Please evaluate them and suggest an assessment and plan.

### Case

HISTORY

**Name:** Mrs. Grace Donovan
**Age:** 81
**Gender:** Female
**Home:** Lives with daughter in a two-story house
**Handed:** Right-handed
**Occupation:** Retired teacher
**Drives:** No, relies on family for transportation
**Activities of daily living:** Independent with meal preparation, needs assistance with bathing
**Past medical history:** Heart failure with reduced ejection fraction (EF 35%), hypertension, ischemic heart disease (stable angina), osteoarthritis
**Regular medications:** Furosemide 40mg daily, Ramipril 5mg daily, Bisoprolol 2.5mg daily, Spironolactone 25mg daily, Atorvastatin 40mg daily, Aspirin 100mg daily
**Allergies:** Codeine (nausea)
**Smoking:** Ex-smoker (quit 20 years ago)
**Alcohol:** None
**Recreational drugs:** Nil
**Over the counter medications:** Paracetamol as needed for joint pain
**Herbal medications:** Nil
**Vaccinations:** Up to date with influenza, pneumococcal, and COVID vaccines
**Sexual history:** Widowed 15 years ago
**Family history:** Father died of myocardial infarction at age 68, mother had osteoporosis

PRESENTING CONCERN

**Chief complaint:** Right Hip Pain After Fall
**History of presenting complaint:** I was in my kitchen making tea this morning when I felt lightheaded and fell onto my right side. I couldn't get up because of severe pain in my right hip. My daughter found me when she came to visit about 30 minutes later. I've been feeling more tired than usual over the past few weeks. My ankles have been swelling more than normal, and I've noticed I need to use more pillows at night to sleep comfortably. I've been taking my water tablets as prescribed, but I've been going to the bathroom less frequently. My appetite has been poor lately, and I find I get full quickly. I've had to stop halfway up the stairs to catch my breath, which isn't normal for me. I haven't had any chest pain recently, but I occasionally feel my heart racing.

NPO STATUS

**Last oral intake:**

- Solid food: Breakfast at 7:00 AM today (toast and tea)
- Clear liquids: Water at 10:00 AM today
  **Duration of fasting:** 5 hours for solids, 2 hours for clear liquids at time of assessment

ANESTHETIC HISTORY

**Previous anesthesia experiences:** General anesthesia for appendectomy (age 35), spinal anesthesia for knee replacement (age 75)
**Complications with anesthesia:** Postoperative confusion after knee surgery
**Family history of anesthetic complications:** None known

SPECIFIC EVALUATION FOR ANESTHESIA

**METs:** Cannot climb half flight of stairs without symptoms

**History of GORD or reflux symptoms:** does not experience any symptoms of reflux

MEDICATION RECONCILIATION

**Specific timing of last dose of medications:**

- Furosemide 40mg: Taken at 8:00 AM today
- Ramipril 5mg: Taken at 8:00 AM today
- Bisoprolol 2.5mg: Taken at 8:00 AM today
- Spironolactone 25mg: Taken at 8:00 AM today

EXAMINATION

**Vital signs:**

- Temperature 36.8°C (98.2°F)
- Heart rate 88 bpm
- Blood pressure 132/78 mmHg
- Respiratory rate 22 breaths per minute
- Oxygen saturations 93% on room air
- Blood glucose level 6.2 mmol/L (112 mg/dL)

AIRWAY ASSESSMENT

**Mallampati classification:** Class II
**Thyromental distance:** 6.5 cm
**Mouth opening and dentition:** Adequate mouth opening (3 finger breadths), full upper dentures, several missing lower teeth
**Neck mobility:** Mildly limited extension
**History of difficult intubation:** None reported

**Mental status:** Alert and oriented to time, place, and person. Glasgow Coma Scale 15/15.
**General inspection:** Elderly female, appears uncomfortable and in pain. Mild peripheral edema noted.

**Cardiovascular examination:**

- Irregular rate and rhythm
- JVP elevated 4cm
- Soft systolic murmur at apex
- Bilateral pitting ankle edema (2+)
- Normal peripheral pulses

**Respiratory examination:** Bilateral basal crackles, otherwise clear
**Abdominal examination:** Soft, mild tenderness in right upper quadrant, no organomegaly
**Neurological examination:**

- Cranial nerves intact
- Power 5/5 in upper limbs, unable to assess lower limbs due to pain
- Normal tone and reflexes
- Plantar responses downgoing

**Musculoskeletal examination:**

- Right lower limb externally rotated and shortened
- Severe pain on minimal movement of right hip
- Tenderness over right greater trochanter

**Height:** 158 cm **Weight:** 64 kg **BMI:** 25.6 kg/m²

INVESTIGATIONS

**Blood tests**

- Complete blood examination: Hemoglobin, blood: 10.6 g/dL [Reference Range: 12.0-15.5 g/dL]
- EUC:
  - Sodium: 133 mmol/L [Reference Range: 135-145 mmol/L]
  - Potassium: 4.8 mmol/L [Reference Range: 3.5-5.0 mmol/L]
  - Creatinine: 1.4 mg/dL (124 μmol/L) [Reference Range: 0.5-1.1 mg/dL]
  - eGFR: 42 mL/min/1.73m² [Reference Range: >60 mL/min/1.73m²]
- Troponin I: 22 ng/L [Reference Range: <16 ng/L]
- BNP: 420 pg/mL [Reference Range: <100 pg/mL]
- Coagulation studies:
  - PT: 13.5 seconds [Reference Range: 11.0-13.5 seconds]
  - INR: 1.1 [Reference Range: 0.8-1.2]
  - aPTT: 34 seconds [Reference Range: 25-35 seconds]
- Urea: 9.2 mmol/L [Reference Range: 2.5-7.8 mmol/L]
- Albumin: 32 g/L [Reference Range: 35-50 g/L]

**Electrophysiology and functional testing**

- ECG: Atrial fibrillation with rate 88 bpm. Poor R wave progression in precordial leads. No acute ST-T wave changes.

**Imaging**

- X-ray right hip: Displaced fracture of the femoral neck, Garden classification IV
- Chest X-ray: Cardiomegaly. Small bilateral pleural effusions. Upper lobe blood diversion.
- Transthoracic Echocardiogram (from 3 months ago): Dilated left ventricle. Reduced left ventricular ejection fraction (35%). Mild-moderate mitral regurgitation. No significant valvular stenosis. No pericardial effusion.

### Key points to elicit

(1) Displaced femoral neck fracture (2) Bibasal lung crepitations, (3) Moderate anemia (4) CXR pleural effusions and upper lobe diversion (5) Reduced eGFR with raised creatinine and urea (6) New atrial fibrillation

### Explanation

This patient presents with a displaced femoral neck fracture following a fall likely precipitated by lightheadedness secondary to decompensated heart failure. Her clinical status indicates volume overload with peripheral edema, elevated JVP, bilateral basal crackles, and cardiomegaly with small pleural effusions on chest X-ray. Her anemia may be contributing to heart failure symptoms and requires evaluation.

The surgical urgency of a hip fracture must be balanced against optimization of her fluid status and cardiac function. Careful fluid management is essential to avoid both pulmonary edema from fluid overload and acute kidney injury from excessive diuresis.

### Assessment and plan exemplar

**Assessment:**
Displaced femoral neck fracture
Decompensated heart failure

Likely acute kidney injury
Moderate anemia
New Atrial fibrillation

ASA Class: IV

**Plan:**

- Strict fluid balance chart with both intake and outputs, daily weights.
- IDC for fluid balance (and in context of #NOF).
- Continue furosemide currently. Noting likely to have periods of fasting, but currently overloaded.
- Consider further afterload reduction if blood pressure exceeds 140mmHg – could consider use a GTN patch for example (avoid nephrotoxic agents)
- Consider atrial fibrillation rate/rhythm control therapy – tachyarrhythmias may further exacerbate her heart failure
- Hold aspirin, rampiril, and atorvastatin. Consider holding bisoprolol
- Anaesthetic consult
- Optimise analgesia – multimodal approach, paracetamol, short acting opioid (SC fentanyl), consider tapentadol, regional anaesthesia with iliacus or femoral block
- Do not give NSAIDs – acute fracture and acute kidney injury
- Consider pre-operative focused bedside TTE for re-evaluation of LV function and the mitral valve
- Serial troponins to monitor for Type 2 NSTEMI
- Consider ICU post-op

### Necessary points for assessment and plan

(1) Displaced femoral neck fracture
(2) Decompensated heart failure
(3) Strict fluid balance
(4) IDC
(5) Anaesthetic consult
(6) Hold aspirin

## Insulin case

### Introduction

Mr. Jack Wallace is a 60-year-old male who has been admitted with a diabetic foot infection requiring debridement. It is currently 11AM. You are part of the ward team managing this patient. Please evaluate them and suggest an assessment and plan.

### Case

HISTORY

Name: Mr. Jack Wallace
Age: 60
Gender: Male
Home: Lives with wife in a two-story house
Handed: Right-handed
Occupation: Retired construction worker
Drives: Yes
Activities of daily living: Independent
Past medical history: Type 2 diabetes mellitus (15 years), hypertension, hyperlipidemia, peripheral neuropathy
Regular medications: Metformin 1000mg twice daily, Insulin glargine at night, Insulin lispro with meals, Lisinopril 10mg daily, Atorvastatin 40mg daily
Allergies: Sulfa drugs (rash)
Smoking: Former smoker (quit 5 years ago), 30 pack-year history
Alcohol: Occasional beer (2-3 standard drinks per week)
Recreational drugs: Nil
Over the counter medications: Multivitamin daily
Herbal medications: Nil
Vaccinations: Up to date with influenza and pneumococcal vaccines
Sexual history: Married 35 years
Family history: Father had type 2 diabetes, mother had hypertension

PRESENTING CONCERN

Chief complaint: Right Foot Pain and Swelling

History of presenting complaint: I noticed a small blister on the bottom of my right foot about two weeks ago after wearing new work boots. I didn't think much of it until it became red and painful about a week ago. Over the last three days, my foot has become increasingly swollen and warm, and I've developed a fever. I've also noticed some discharge from the wound. My blood sugars have been running high lately, between 200-350 mg/dL (11.1-19.4 mmol/L), despite adjusting my insulin. I've been having trouble keeping up with my regular insulin regimen because of my work schedule. I usually take Metformin twice daily and insulin glargine at night with insulin lispro before meals, but I often forget my lunchtime dose. I've been feeling more tired than usual and have had decreased appetite. I haven't been checking my feet regularly as recommended by my doctor.

NPO STATUS

Last oral intake:

- Solid food: Breakfast at 7:00 AM today (toast and coffee)
- Clear liquids: Water at 10:00 AM today
- Duration of fasting: 4 hours for solids, 1 hour for clear liquids at time of assessment

ANESTHETIC HISTORY

**Previous anesthesia experiences:** General anesthesia for appendectomy (age 25), local anesthesia for dental procedures

**Complications with anesthesia:** Post-operative nausea after appendectomy, no other complications

**Family history of anesthetic complications:** None known

SPECIFIC EVALUATION FOR ANESTHESIA

**Metabolic equivalents (METs) capacity:** can climb one flight of stairs without symptoms

**History of GORD or reflux symptoms:** experiences GORD on occasions, usually after a large fatty meal, takes some over the counter ant-acids but does not see his GP for this

MEDICATION RECONCILIATION

Specific timing of last dose of medications:

- Metformin 1000mg: Taken at 8:00 AM today
- Insulin glargine: 30 units at 10:00 PM yesterday
- Insulin lispro: 8 units at 7:00 AM today
- Lisinopril 10mg: Taken at 8:00 AM today

EXAMINATION

Vital signs:

- Temperature 38.2°C (100.8°F)
- Heart rate 102 bpm
- Blood pressure 145/90 mmHg
- Respiratory rate 18 breaths per minute
- Oxygen saturations 96% on room air
- Blood glucose level 285 mg/dL (15.8 mmol/L)

AIRWAY ASSESSMENT

Mallampati classification: Class II

Thyromental distance: 7 cm

Mouth opening and dentition: Adequate mouth opening (3 finger breadths), good dentition with few missing teeth

Neck mobility: Full range of motion

History of difficult intubation: None reported

Mental status: Alert and oriented to time, place, and person. Glasgow Coma Scale 15/15. General inspection: Middle-aged male, appears uncomfortable and in pain. Mild diaphoresis noted.

Cardiovascular examination:

- Regular rate and rhythm
- JVP not elevated
- Normal heart sounds
- No murmurs, rubs, or gallops
- Peripheral pulses diminished in bilateral lower extremities
- Capillary refill >4 seconds in right foot, 3 seconds in left foot

Respiratory examination: Clear to auscultation bilaterally

Abdominal examination: Soft, non-tender, no organomegaly

Neurological examination:

- Cranial nerves intact
- Power 5/5 in all limbs
- Diminished sensation to light touch and pinprick in stocking distribution bilaterally
- Absent ankle reflexes bilaterally
- No cerebellar signs

Musculoskeletal examination:

- Right foot with 5 cm erythematous, warm, fluctuant area on plantar surface
- 2 cm ulcer with purulent discharge at base of right first metatarsal
- Surrounding cellulitis extending to mid-foot
- Dorsal foot edema
- Left foot with callus formation but no active ulceration

Height: 175 cm Weight: 92 kg BMI: 30.0 kg/m²

INVESTIGATIONS

Blood tests

- Complete blood examination:
  - Hemoglobin: 11.5 g/dL [Reference Range: 13.5-17.5 g/dL]
  - White blood cell count: 16.5 × 10^9/L [Reference Range: 4.0-11.0 × 10^9/L]
  - Neutrophil count: 13.2 × 10^9/L [Reference Range: 1.8-7.5 × 10^9/L]
- EUC:
  - Sodium: 138 mmol/L [Reference Range: 135-145 mmol/L]
  - Potassium: 4.2 mmol/L [Reference Range: 3.5-5.0 mmol/L]
  - Creatinine: 1.3 mg/dL (115 μmol/L) [Reference Range: 0.7-1.2 mg/dL]
  - eGFR: 58 mL/min/1.73m² [Reference Range: >90 mL/min/1.73m²]
- CRP: 85 mg/L [Reference Range: <5 mg/L]
- HbA1c: 9.8% [Reference Range: <7.0% for diabetics]
- Blood cultures: Pending
- Wound culture: Pending
- Ketone: 0.4 [Reference Range: <0.6]
- VBG:
  - pH 7.35 [Reference Range: 7.35-7.45]
  - CO2: 40 [Reference Range: 35-45]
  - HCO3: 22
  - Lactate: 2.0 [Reference Range: <1.0]

Imaging

- X-ray right foot: No evidence of osteomyelitis. Soft tissue swelling present.
- MRI right foot: Cellulitis with 3 cm x 2 cm abscess at the base of the first metatarsal. No definitive evidence of osteomyelitis.

### Key points to elicit

(1) Diabetic foot infection with abscess
(2) Poor glycemic control (HbA1c 9.8%)
(3) Leukocytosis (WBC 16.5)
(4) Mild anemia (Hgb 11.5 g/dL)
(5) Stage 3 CKD (eGFR 58)

### Explanation

This patient presents with a diabetic foot infection requiring surgical debridement, complicated by poor glycemic control. His elevated HbA1c of 9.8% indicates chronic hyperglycemia, and his current blood glucose of 285 mg/dL suggests ongoing poor control. The infection is evidenced by leukocytosis, fever, and clinical signs of inflammation with abscess formation confirmed on MRI. The patient has multiple diabetic complications including peripheral neuropathy and stage 3 chronic kidney disease, which will influence his perioperative management.

The surgical urgency of diabetic foot infection must be balanced against optimization of his glycemic control. Prompt surgical intervention is necessary to control the infection and prevent further tissue damage, while careful insulin management is essential to maintain glycemic control perioperatively and reduce the risk of complications.

### Assessment and plan exemplar

Assessment:

- Diabetic foot infection with abscess
- Poorly controlled type 2 diabetes mellitus
- Peripheral neuropathy
- Stage 3 CKD
- ASA Class: III

Plan:

- IV antibiotics – follow local protocols. Choices may include: amoxicillin-clavulanic acid OR cefazolin + metronidazole OR Piperacillin-tazobactam +/- clindamycin +/- vancomycin (pending risk of MRSA or concern of necrotising fasciitis or degree of septic shock)
- Fluid resuscitation – up to 30ml/kg = 2.7L
- Planning for surgical debridement
- Anaesthetic consult
- Convert to IV insulin protocol aiming BGL 6-10 – BGL management as per protocol and monitor ketones 6 hourly
- Hold regular insulin agents whilst on the insulin infusion – aim to restart once recovered from surgery
- Hold Metformin and lisinopril – both can cause complications in the perioperative unwell patient
- QID BGLs once off insulin infusion
- Endocrine consult + diabetic nurse educator

### Necessary points for assessment and plan

(1) Diabetic foot infection requiring debridement
(2) Uncontrolled diabetes
(3) IV insulin protocol
(4) Frequent glucose monitoring
(5) Antibiotic therapy

## NBM medications case

### Introduction

Mr. Gareth Monahan is a 75-year-old male admitted to the emergency department with abdominal pain and vomiting, later diagnosed with a perforated small bowel obstruction requiring surgical intervention. You are part of the ward team managing this patient. Please evaluate them and suggest an assessment and plan.

### Case

HISTORY

Name: Mr. Gareth Monahan
Age: 75
Gender: Male
Home: Lives with wife in a two-story house
Handed: Right-handed
Occupation: Retired accountant
Drives: Yes, but mostly for short distances
Activities of daily living: Independent with some mobility limitations
Past medical history: Hypertension (diagnosed 10 years ago), Parkinson's disease (diagnosed 5 years ago), Epilepsy (since age 15), Appendectomy (age 40)
Regular medications: Madopar 100/25mg TDS (0800, 1400, 2000), Sodium valproate 700mg BD, Amlodipine 5mg daily, Ramipril 5mg daily
Allergies: None known
Smoking: Former smoker (quit 20 years ago)
Alcohol: Occasional (2-3 standard drinks per week)
Recreational drugs: Nil
Over the counter medications: Paracetamol PRN for joint pain
Herbal medications: Nil
Vaccinations: Influenza (3 months ago), Pneumococcal (2 years ago)
Sexual history: Married 45 years
Family history: Father had colon cancer at age 70

PRESENTING CONCERN

Chief complaint: Moderate to Severe Abdominal Pain and Vomiting

History of presenting complaint: I started having abdominal pain two days ago. It was initially around my belly button but now it's more on the right side and getting worse. I've vomited about 5 times in the last 24 hours, and haven't been able to keep much down. The vomit was initially food, then yellow-green. I haven't had a bowel movement for 2 days, and my stomach feels bloated. The pain is constant, about 7/10 in intensity. I feel a bit weak when I stand up for too long. My wife drove me to the hospital when the pain became more severe this morning. I've had mild tremors from my Parkinson's that seem slightly worse today, probably because I missed my morning medication. I've never had pain like this before, though I did have my appendix removed many years ago.

NPO STATUS

Last oral intake:

- Solid food: Light dinner yesterday (soup) at 6:00 PM
- Clear liquids: Small sips of water 3 hours ago
  Duration of fasting: 16 hours for solids, 3 hours for clear liquids at time of assessment

ANESTHETIC HISTORY

Previous anesthesia experiences: General anesthesia for appendectomy (age 40)
Complications with anesthesia: Postoperative nausea and vomiting
Family history of anesthetic complications: None known

SPECIFIC EVALUATION FOR ANESTHESIA

Metabolic equivalents (METs) capacity: Can climb one flight of stairs with minimal rest

MEDICATION RECONCILIATION

Specific timing of last dose of medications:

- Madopar 100/25mg: Missed morning dose (due 8:00 AM today)
- Sodium valproate 700mg: Last dose 10:00 PM yesterday
- Amlodipine 5mg: Last dose 8:00 AM yesterday
- Ramipril 5mg: Last dose 8:00 AM yesterday

EXAMINATION

Vital signs:

- Temperature 37.9°C (100.2°F)
- Heart rate 102 bpm
- Blood pressure 110/70 mmHg
- Respiratory rate 20 breaths per minute
- Oxygen saturations 96% on room air
- Blood glucose level 135 mg/dL (7.5 mmol/L)

AIRWAY ASSESSMENT

Mallampati classification: Class II
Thyromental distance: 7 cm
Mouth opening and dentition: Adequate mouth opening (3 finger breadths), complete dentition with dental crowns on upper molars
Neck mobility: Good range of motion
History of difficult intubation: None reported

Mental status: Alert and oriented to time, place, and person. Glasgow Coma Scale 15/15.
General inspection: Elderly male, appears uncomfortable and in moderate distress. Mildly dehydrated.

Cardiovascular examination:

- Mildly tachycardic, regular rhythm
- JVP not elevated
- Heart sounds normal, no murmurs
- Capillary refill time 2 seconds
- Peripheral pulses present and normal

Respiratory examination: Clear breath sounds throughout

Abdominal examination:

- Moderately distended
- Generalized tenderness with localized guarding in right lower quadrant
- Hypoactive bowel sounds
- No palpable masses
- Percussion note tympanic centrally
- Old appendectomy scar right lower quadrant

Neurological examination:

- Cranial nerves intact
- Mild resting tremor in right hand
- Mild cogwheel rigidity in right upper limb
- Power 5/5 all limbs
- Normal reflexes
- Plantar responses downgoing

Height: 175 cm Weight: 72 kg BMI: 23.5 kg/m²

INVESTIGATIONS

Blood tests

- Complete blood examination:
  - White cell count: 15.2 × 10^9/L [Reference Range: 4.0-11.0 × 10^9/L]
  - Hemoglobin: 13.5 g/dL [Reference Range: 13.5-17.5 g/dL]
  - Platelets: 350 × 10^9/L [Reference Range: 150-400 × 10^9/L]
- EUC:
  - Sodium: 134 mmol/L [Reference Range: 135-145 mmol/L]
  - Potassium: 3.6 mmol/L [Reference Range: 3.5-5.0 mmol/L]
  - Chloride: 98 mmol/L [Reference Range: 98-108 mmol/L]
  - Bicarbonate: 22 mmol/L [Reference Range: 22-29 mmol/L]
  - Urea: 8.5 mmol/L [Reference Range: 2.5-7.5 mmol/L]
  - Creatinine: 1.2 mg/dL (106 μmol/L) [Reference Range: 0.7-1.2 mg/dL]
- LFTs:
  - Albumin: 35 g/L [Reference Range: 35-50 g/L]
  - ALT: 42 U/L [Reference Range: <45 U/L]
  - ALP: 75 U/L [Reference Range: 30-110 U/L]
  - Bilirubin: 1.0 mg/dL [Reference Range: <1.2 mg/dL]
- CRP: 120 mg/L [Reference Range: <5 mg/L]
- Lactate: 2.5 mmol/L [Reference Range: 0.5-2.2 mmol/L]
- Coagulation studies:
  - PT: 13.2 seconds [Reference Range: 11.0-13.5 seconds]
  - INR: 1.1 [Reference Range: 0.8-1.2]
  - aPTT: 33 seconds [Reference Range: 25-35 seconds]
- Valproate level: 65 μg/mL [Reference Range: 50-100 μg/mL]

Electrophysiology and functional testing

- ECG: Sinus tachycardia at 102 bpm. No ST-T wave changes.

Imaging

- Abdominal X-ray: Dilated loops of small bowel with air-fluid levels. Small amount of free air under right hemidiaphragm.
- CT abdomen/pelvis: Moderately dilated small bowel loops with transition point in distal ileum. Small amount of free fluid and localized free intraperitoneal air. Small bowel wall thickening. Mesenteric inflammatory changes.

### Key points to elicit

(1) Perforated small bowel obstruction (2) Mild leukocytosis, elevated CRP (3) Borderline hemodynamic stability (4) Mild dehydration (5) Parkinson's disease

### Explanation

This patient presents with a perforated small bowel obstruction requiring urgent surgical intervention. The history of progressive abdominal pain, vomiting, and reduced bowel movements is consistent with bowel obstruction. The presence of mild fever, tachycardia, and laboratory findings of leukocytosis and elevated inflammatory markers indicates an inflammatory response to the perforation, confirmed by free air on imaging studies.

The patient's comorbidities, particularly Parkinson's disease and epilepsy, require careful medication management in the perioperative period. Missing Madopar doses can worsen Parkinson's symptoms, while maintaining therapeutic valproate levels is essential to prevent seizures. The patient shows signs of mild dehydration but is hemodynamically stable, requiring careful fluid resuscitation.

Urgent surgical intervention is needed, but adequate preoperative optimization should be prioritized to reduce perioperative morbidity.

### Assessment and plan exemplar

Assessment:
Perforated small bowel obstruction
Mild systemic inflammatory response
Parkinson's disease and Epilepsy medications in setting of nil by mouth

Plan:

- IV fluid resuscitation – up to 30ml/kg
- IV antibiotics – follow local protocols, general advice for perforated viscus with evidence of peritonitis is amoxicillin + metronidazole + gentamycin
- Urgent surgical exploration
- Analgesia – IV paracetamol +/- non-oral opioid therapy for acute pain  noting this may be a feature of worsening disease progression and should trigger escalation of definitive management
- NBM – consider maintenance IV fluid therapy after initial resuscitation
- Discuss Parkinson’s medications with pharmacy and/or neurology. Since NBM and 300mg total daily levodopa dose, likely rotigotine 10mg/24h. Aiming to recommence as soon as possible, including via NGT.
- Sodium valproate continue 700mg IV BD (1:1 conversion)
- Hold antihypertensives currently
- Consider underlying cause of obstruction and work-up accordingly

### Necessary points for assessment and plan

(1) Perforated bowel obstruction
(2) Fluid resuscitation
(3) Antibiotic therapy
(4) Surgical intervention
(5) Parkinson’s medication addressed (either discussing for help
(6) Continue sodium valproate IV

## Antibiotic prophylaxis case

### Introduction

Layla Ahmed is a 29-year-old female who has been admitted to the emergency department following a motorcycle accident resulting in a comminuted distal tibia-fibula fracture requiring surgical intervention. It is 3:30PM. You are part of the ward team managing this patient. Please evaluate them and suggest an assessment and plan.

### Case

HISTORY

**Name:** Layla Ahmed
**Age:** 29
**Gender:** Female
**Home:** Lives with roommate in a second-floor apartment
**Handed:** Right-handed
**Occupation:** Graphic designer
**Drives:** Yes, owns a motorcycle
**Activities of daily living:** Independent
**Past medical history:** Seasonal allergies, appendectomy (age 15)
**Regular medications:** Loratadine 10mg daily (seasonal)
**Allergies:** Amoxicillin (anaphylaxis), adhesive tape (skin irritation)
**Smoking:** Social smoker (2-3 cigarettes weekly)
**Alcohol:** Moderate (4-6 standard drinks per week)
**Recreational drugs:** Denies
**Over the counter medications:** Occasional ibuprofen for headaches
**Herbal medications:** Nil
**Vaccinations:** Up to date including tetanus
**Sexual history:** Sexually active, uses contraception
**Family history:** Mother with hypothyroidism, father with hypertension

PRESENTING CONCERN

**Chief complaint:** Right Ankle Pain and Deformity After Motorcycle Accident

**History of presenting complaint:** I was riding my motorcycle when a car suddenly pulled out in front of me. I swerved to avoid it and crashed into a guardrail. My right leg took the impact and was trapped between the motorcycle and the guardrail. I felt immediate severe pain and noticed obvious deformity of my ankle. Bystanders called an ambulance. I've had no loss of consciousness, no other injuries besides some minor abrasions on my right arm. I can feel and move my toes but it's extremely painful. I've had nothing to eat or drink since the accident about 2 hours ago (last liquids were this morning at 07:30AM, 8 hours ago). I had a severe allergic reaction to amoxicillin when I was 16 - my throat closed up and I needed epinephrine.

NPO STATUS

**Last oral intake:**

- Solid food: Breakfast at 7:30 AM (toast and coffee)
- Clear liquids: Coffee at 7:30 AM **Duration of fasting:** 8 hours for solids and liquids at time of assessment

ANESTHETIC HISTORY

**Previous anesthesia experiences:** General anesthesia for appendectomy (age 15) **Complications with anesthesia:** Post-operative nausea, otherwise uncomplicated

**Family history of anesthetic complications:** None known

SPECIFIC EVALUATION FOR ANESTHESIA

**Metabolic equivalents (METs) capacity:** Can run short distances, exercise regularly (>4 METs)

MEDICATION RECONCILIATION

**Specific timing of last dose of medications:**

- Loratadine 10mg: Taken at 7:00 AM today
- Ibuprofen 400mg: Taken 3 days ago for headache

EXAMINATION

**Vital signs:**

- Temperature 37.2°C (99.0°F)
- Heart rate 110 bpm
- Blood pressure 130/85 mmHg
- Respiratory rate 18 breaths per minute
- Oxygen saturations 98% on room air

AIRWAY ASSESSMENT

**Mallampati classification:** Class I

**Thyromental distance:** 7 cm

**Mouth opening and dentition:** Full mouth opening (3+ finger breadths), healthy dentition **Neck mobility:** Normal range of motion

**History of difficult intubation:** None reported

**Mental status:** Alert and oriented to time, place, and person. Glasgow Coma Scale 15/15. **General inspection:** Young female in obvious distress, holding right lower limb immobile.

**Cardiovascular examination:**

- Regular rate and rhythm
- Normal heart sounds
- No murmurs
- Capillary refill <2 seconds
- Strong peripheral pulses in unaffected limbs

**Respiratory examination:** Clear to auscultation bilaterally

**Abdominal examination:** Soft, non-tender, appendectomy scar in right lower quadrant

**Neurological examination:**

- Cranial nerves intact
- Power 5/5 in all limbs except right lower limb
- Intact sensation in right foot
- Diminished but present dorsalis pedis and posterior tibial pulses in right foot

**Musculoskeletal examination:**

- Right lower leg with obvious deformity at ankle
- Significant swelling and ecchymosis
- Skin intact but tense
- Extreme tenderness to palpation
- Limited range of motion due to pain
- Neurovascular status intact distally

**Height:** 168 cm **Weight:** 65 kg **BMI:** 23.0 kg/m²

INVESTIGATIONS

**Blood tests**

- Complete blood examination:
  - Hemoglobin 13.2 g/dL [Reference Range: 12.0-15.5 g/dL]
  - WBC: 12.5 x 10^9/L [Reference Range: 4.0-11.0 x 10^9/L]
  - Platelets: 245 x 10^9/L [Reference Range: 150-400 x 10^9/L]
- EUC:
  - Creatinine: 0.8 mg/dL [Reference Range: 0.5-1.1 mg/dL]
  - Glucose: 110 mg/dL [Reference Range: 70-100 mg/dL]
- Coagulation studies:
  - PT: 12.5 seconds [Reference Range: 11.0-13.5 seconds]
  - INR: 1.0 [Reference Range: 0.8-1.2]
  - aPTT: 30 seconds [Reference Range: 25-35 seconds]

**Imaging**

- X-ray right ankle and leg: Comminuted distal tibia-fibula fracture with intra-articular extension
- CT scan: Confirms comminuted fracture with multiple fragments, significant displacement, and ankle joint involvement
- Chest X-ray: Normal cardiac silhouette. Clear lung fields.

### Key points to elicit

(1) Comminuted distal tibia-fibula fracture (2) Anaphylaxis to amoxicillin (3) Requires surgical fixation (4) closed fracture (5) Neurovascular status intact

### Explanation

This patient presents with a traumatic comminuted distal tibia-fibula fracture requiring surgical intervention. The most significant anesthetic concern is her history of anaphylaxis to amoxicillin, which necessitates careful antibiotic selection for surgical prophylaxis. Perioperative antibiotic prophylaxis is critical to prevent surgical site infection, particularly in orthopedic cases involving implanted hardware. Given her severe allergy to amoxicillin, an appropriate alternative antibiotic regimen must be selected that provides adequate coverage against common orthopedic wound pathogens including Staphylococcus aureus.

### Assessment and plan exemplar

**Assessment:**

Comminuted distal tibia-fibula fracture

Amoxicillin anaphylaxis

ASA Class: II

**Plan:**

- NPO status planning for surgical fixation
- Appropriate alternative antibiotic prophylaxis, review local protocols likely clindamycin or vancomycin
- Analgesia, including regular paracetamol and PRN opioid – note that escalating pain or analgesic requirement may be an indicator of increasing compartment pressures, and regular assessment for compartment syndrome is warranted
- Anaesthetics consult including consider regional anesthesia
- Q1H neurovascular observations until successful reduction

### Necessary points for assessment and plan

(1) Comminuted distal tibia-fibula fracture (2) Amoxicillin anaphylaxis (3) Alternative antibiotic selection (4) Timing of antibiotic administration (5) Regional anesthesia consideration

# **Supplementary Information 3** – LLM Responses not in screenplays

| **Case** | **Question** | **Answer** | **Category** |
| --- | --- | --- | --- |
| Perioperative anticoagulation | What about today? | Today, I still feel nauseated but haven't vomited again. | History (nausea and vomiting) |
|  | How many years did you smoke for? | I smoked for about 20 years before quitting. | History (smoking) |
|  | When did you last take empagliflozin and metformin? | I last took Empagliflozin and Metformin at 8:00 PM yesterday. | History (medication timing) |
|  | Can you climb 2 flights of stairs without getting short of breath? | I can climb one flight of stairs without symptoms, but not two. | History (METS) |
|  | Do you feel light headed or dizzy? | No, I haven't experienced any light-headedness or dizziness. | History (cardiovascular symptoms) |
|  | Any caps or crowns? | I don't have any dental caps or crowns. | History (dental) |
|  | did post-op nausea settle with administration of antiemetics | I’m not sure, as it was a long time ago. I don’t remember the treatment details. | History (anaesthetic comoplications) |
|  | do you see a cardiologist | No, I do not see a cardiologist regularly. My general practitioner manages my atrial fibrillation. | History (Specialist management) |
|  | any recent echo | I haven't had an echocardiogram in recent months. My last one was over a year ago. | History (Cardiovascular investigations) |
|  | what is usual BSL | I usually keep my blood sugar levels between 100 and 150 mg/dL. | History (T2DM control) |
|  | if given antiemetics did psot-op nausea and vomiting get better | Yes, the postoperative nausea improved with antiemetics after my appendectomy. | History (anaesthetic complications) |
|  | echo | The echocardiogram has been requested and is pending. | Investigation (imaging) |
|  | What do you take apixaban for | I take apixaban for atrial fibrillation. | History (medication indication) |
|  | Any respiratory disease? | No, I don't have any known respiratory diseases. | History (PMHx) |
|  | Have you had any medications for pain | No, I haven't taken any medications for pain recently. | History (OTC medications) |
|  | CT abdo pelvis | CT abdomen pelvis has been requested and is pending. | Investigation (imaging) |
|  | how much did you smoke for and for how long | I smoked for about 20 years before quitting 15 years ago. | History (smoking) |
|  | was it a pack a day | I smoked about half a pack a day. | History (smoking) |
|  | how do you manage your diabetes | I manage my diabetes with Metformin and Empagliflozin, along with a healthy diet and regular monitoring. | History (diet and exercise) |
|  | in regards to your AF, have you ever had a stroke | No, I have never had a stroke. | History (PMHx) |
|  | have you ever had a heart attack | No, I have never had a heart attack. | History (PMHx) |
|  | do you have any respiratory conditions. asthma, COPD or OSA | No, I do not have any respiratory conditions like asthma, COPD, or OSA. | History (PMHx) |
|  | magnesium | This investigation has been requested and is pending. | Investigation (bloods) |
|  | CT abdomen | This investigation has been requested and is pending. | Investigation (imaging) |
|  | abg | This information has been requested and is pending. | Investigation (bloods) |
| Perioperative fluid balance | Nausea or vomiting | I haven't experienced any nausea or vomiting recently, except with codeine. | History (nausea and vomiting) |
|  | Any previous lightheadedness | No, I haven't experienced lightheadedness before this incident. | History (cardiovascular symptoms) |
|  | Fevers? | I haven't experienced any fevers. | History (infectious symptoms) |
|  | Cough? | I have not had any cough. | History (infectious symptoms) |
|  | Dysuria | I haven't experienced any dysuria. | History (infectious symptoms) |
|  | EUC, CBE, CRP, Coags, BGL, G+H | EUC, CBE, and coagulation results have been provided. CRP and G+H have been requested and are pending. BGL is 6.2 mmol/L (112 mg/dL). | Investigation (bloods) |
|  | PLT | Platelet count has been requested and is pending. | Investigation (bloods) |
|  | CTB | CT brain has been requested and is pending. | Investigations (imaging) |
|  | any bleeding | No, I haven't noticed any bleeding. | History (trauma symptoms) |
|  | any lung disease | I don't have a history of lung disease. | History (PMHx) |
|  | bsl | Blood glucose level has been requested and is pending. | Investigation (bloods) |
|  | glucose | Blood glucose level has been requested and is pending. | Investigation (bloods) |
|  | lfts | Liver function tests have been requested and are pending. | Investigation (bloods) |
|  | ck | Creatine kinase level has been requested and is pending. | Investigation (bloods) |
|  | Do you see a cardiologist regularly | I do not have regular follow-ups with a cardiologist. | History (Specialist management) |
|  | How bad is your pain currently out of 10 | My pain is about 8 out of 10 right now. | History (pain) |
|  | group and hold | Group and hold has been requested and is pending. | Investigation (bloods) |
|  | has anyone done a nerve block yet | No, I haven't had a nerve block yet. | Management (analgesia) |
|  | who manages your heart | My heart conditions are managed by my cardiologist. | History (Specialist management) |
|  | when did you last see them | I can't remember the exact date of my last appointment with my cardiologist. | History (Specialist management) |
|  | is your cardiologist happy with your heart | Generally, my cardiologist has been monitoring my conditions, but recent symptoms have raised concerns. | History (Specialist management) |
|  | have you had stents or bypass surgery on the heart | No, I have not had stents or bypass surgery on my heart. | History (PMHx) |
|  | any lungs conditions | I do not have any known lung conditions. | History (PMHx) |
|  | latest angiogram | The latest angiogram has been requested and is pending. | Investigations (Angiogram) |
|  | CTPA | CTPA has been requested and is pending. | Investigations (imaging) |
|  | ABG | ABG has been requested and is pending. | Investigation (bloods) |
|  | vbg | VBG has been requested and is pending. | Investigation (bloods) |
| Case 496 – Perioperative antibiotics | Reflux | I haven't experienced any reflux or gastrointestinal issues. | History (gord) |
|  | reduction in ED? | I haven't received any pain relief or reduction in the emergency department yet. | Management (analgesia) |
|  | LFTs | LFTs have been requested and are pending. | Investigation (bloods) |
|  | Have you ever had cephalexin or cephazolin | I haven't taken either cephalexin or cephazolin before. | History (allergies) |
|  | Have you had allergy testing before? | No, I haven't had allergy testing before. | History (allergies) |
|  | Have you had any pain relief | No, I haven't received any pain relief since the accident. | Management (analgesia) |
|  | ECG | Requested and is pending. | investigations (ECG) |
|  | Group and hold | Requested and is pending. | Investigation (bloods) |
|  | have you had pain relief so far | No, I haven't had any pain relief since the accident. | Management (analgesia) |
|  | do you get reflux | No, I don’t experience reflux. | History (gord) |
|  | any heart or lung conditions | No, I do not have any heart or lung conditions. | History (PMHx) |
| Case 497 – Perioperative nil by mouth | CBE, EUC, LFTs, Group and hold | CBE and EUC results provided; LFTs show normal liver function; Group and hold test has been requested and is pending. | Investigation (bloods) |
|  | CXR | CXR has been requested and is pending. | Investigations (imaging) |
|  | are you still feeling nauseous | Yes, I'm still feeling very nauseous. | History (nausea and vomiting) |
|  | when did you last take your medications | I missed my morning dose of Madopar today; last doses of others were yesterday evening and this morning. | History (medication timing) |
|  | chest xray | This investigation has been requested and is pending. | Investigation (imaging) |
|  | when did you last vomit | I last vomited about an hour ago. | History (nausea and vomiting) |
|  | any fevers, dizziness, shortness of breath or chcest pain | No fevers, dizziness, shortness of breath, or chest pain. | History (infectious symptoms) |
|  | what is madopar | Madopar is a medication used to treat symptoms of Parkinson's disease. | History (medication indication) |
|  | do you have any autonomic dysfunction from parkinsons | I haven't noticed significant autonomic dysfunction from my Parkinson's. | History (PMHx) |
|  | vbg | It has been requested and is pending. | Investigation (bloods) |
|  | cxr | It has been requested and is pending. | Investigation (imaging) |
| Case 498 – Perioperative glucose | ECG | ECG has been requested and is pending. | investigations (ECG) |
|  | LFTs | It has been requested and is pending. | Investigation (bloods) |
|  | What doses of insulin do you use | I use 30 units of insulin glargine at night and 8 units of insulin lispro with meals. | History (medication dose) |
|  | Have you noticed any changes to your urination | I haven't noticed any significant changes to my urination recently. | history (urinary symptoms) |
|  | hows is your breathing, including at night, any shortness of breath or snoring or osa | My breathing is fine; no shortness of breath, snoring, or symptoms of obstructive sleep apnea. | History (respiratory symptoms) |
